# Mobile patch EEGs – practicalities, acceptability, barriers and facilitators to patch use in people with mild or moderate dementia

**DOI:** 10.1002/alz70861_108041

**Published:** 2025-12-23

**Authors:** Gill Livingston, Penny Rapaport, Yuto Satake, Andrew Sommerlad, Tsuyoshi Sekitani, Ruan‐Ching Yu, Teppei Araki, Monica Manela, Clare Yu, Yuki Miyazaki, Masahiro Hata, Manabu Ikeda

**Affiliations:** ^1^ Psychiatry, University College London, London, London UK; ^2^ University College London, London UK; ^3^ Osaka University Graduate School of Medicine, Suita‐shi Japan; ^4^ UCL, London, London UK; ^5^ University College London, London, London UK; ^6^ Osaka University, Osaka, Osaka Japan; ^7^ Osaka University, Osaka Japan; ^8^ Osaka University Graduate School of Medicine, Suita, Osaka Japan

## Abstract

**Background:**

People with dementia commonly have sleep disturbance which negatively impacts mental and physical functioning. Family carers of people with dementia struggle to cope with relatives’ sleep disturbance and this may lead to care home admission. So sleep problems increase dementias’ individual and societal costs. We developed the intervention, Dementia Related Manual for Sleep ‐ Strategies for Relatives (DREAMS‐START), incorporating light, routine, activity, behavioural management and relaxation for sleep disturbances in this group. Validated carer report measures found DREAMS‐START improved people with dementia and their family carers’ sleep at 4 and 8 months.

We wanted to use actigraphy; biologically inferring sleep from movement to enable investigators and participants to rapidly understand what works for them in our intervention, enhancing personalisation. However, many people with dementia would not wear wrist word accelerometers. We tried EEG headbands worn at home, but they were unacceptably heavy, uncomfortable and disturbed sleep.

**Method:**

Prof Sekitani’s team in Japan have developed a flexible sheet sensor that stick to the forehead, which is transparent and stretchable, and wirelessly records EEGs. This UCL/Osaka international collaboration is assessing it for feasibility and acceptability to record overnight sleep in people with dementia who have decisional capacity.

**Result:**

It is complex to measure sleep biologically in people living with dementia with important practical, ethical and acceptability considerations.

**Conclusion:**

This work enables us to build future work to understand and improve sleep for people with dementia.

